# Electrodynamics of Topologically Ordered Quantum Phases in Dirac Materials

**DOI:** 10.3390/nano11112914

**Published:** 2021-10-30

**Authors:** Musa A. M. Hussien, Aniekan Magnus Ukpong

**Affiliations:** Theoretical and Computational Condensed Matter and Materials Physics Group, School of Chemistry and Physics, College of Agriculture, Engineering and Science, University of KwaZulu-Natal, Pietermaritzburg 3201, South Africa; 219042924@stu.ukzn.ac.za

**Keywords:** topological quantum phase transitions, collective excitation, nanoline, charge density wave, Chern number

## Abstract

First-principles calculations of the electronic ground state in tantalum arsenide are combined with tight-binding calculations of the field dependence of its transport model equivalent on the graphene monolayer to study the emergence of topologically ordered quantum states, and to obtain topological phase diagrams. Our calculations include the degrees of freedom for nuclear, electronic, and photonic interactions explicitly within the quasistatic approximation to the time-propagation-dependent density functional theory. This field-theoretic approach allows us to determine the non-linear response of the ground state density matrix to the applied electromagnetic field at distinct quantum phase transition points. Our results suggest the existence of a facile electronic switch between trivial and topologically ordered quantum states that may be realizable through the application of a perpendicular electric or magnetic field alongside a staggered-sublattice potential in the underlying lattice. Signatures of the near field electrodynamics in nanoclusters show the formation of a quantum fluid phase at the topological quantum phase transition points. The emergent carrier density wave transport phase is discussed to show that transmission through the collective excitation mode in multilayer heterostructures is a unique possibility in plasmonic, optoelectronic, and photonic applications when atomic clusters of Dirac materials are integrated within nanostructures, as patterned or continuous surfaces.

## 1. Introduction

One of the key drivers of emerging technologies is the ability to deliver dissipation-free transport of carriers over topologically protected quantum states. On the other hand, it is also technologically attractive to use the topological states of photonic crystals to realize lossless waveguides for optical communication. As such, the search for topologically ordered electronic phases of quantum matter is an active research frontier [1]. Topological protection of carrier transport is required in applications like spintronics, plasmonics, high-density data storage, and tunneling field-effect transistors, etc. and Dirac materials [2] are promising candidates for delivering such quantum states. This is because their monolayers can be incorporated into metamaterials and multilayer heterostructures to guarantee the existence of chiral edge states. The major feature of a topologically protected electronic phase is the non-trivial edge state, which is robust against all perturbations including long-ranged interactions [3,4,5,6,7] and conserved on graphene lattice by bulk-edge correspondence (BEC) principle [8].

The electronic ground state in bulk tantalum arsenide (TaAs) is determined here from first principles and combined with tight-binding calculations of the external field dependence of the low-energy band dispersion in graphene to study the emergence of topologically ordered quantum states in the carrier transport model of Dirac materials. The evolution of graphene band structure with changes in the topological order parameters is employed here to study the dependence of the magnitude of the emergent bandgap on-field tuning parameters. These emergent quantum transport phases are practically realizable using engineered multilayer material platforms when Dirac materials are incorporated into stacked multilayer heterostructures in their 2D or monolayer form. This is because their chiral edge and surface electron states are topologically protected against perturbations, thus permitting topological spintronics and optoelectronics.

Achieving carrier transport through topologically protected electron states is becoming the desirable strategy for developing materials for emerging technologies because the continued scaling era is changing rapidly to the era of hyper-scaling [9]. We argue here that this is achievable when 2D forms of Dirac materials that host topologically protected edge states are integrated within vertically stacked van der Waals multilayer heterostructures [10,11,12,13]. This is because the presence of chiral anomalies in the spin conductance spectra of heterostructures makes carrier transport field-tunable [12]. However, a different problem emerges from the coexistence of bulk and topological electron states due to the presence of intrinsic long-ranged disorder in metamaterials and multilayer heterostructures [14]. By contrast, magnetotransport experiments on bulk Sb_2_Te_3_ show a weak mixing between the surface and bulk electron states [15]. The weak coupling is due to the absence of long-range disorder in bulk Sb_2_Te_3_. Thus, the long-ranged disorder in multilayer heterostructures means that alternative strategies for using heterostructures as the platform for realizing coherent current must be developed.

Herein, we identify such strategies by developing a rational understanding of the topological electron states and their topological quantum phase transitions (TQPTs) for emerging applications in photonics, optoelectronics, and spintronics. We present combined first principles and field-theoretic calculations of the electrodynamic signatures of carriers at characteristic energies at which distinct topological phase transitions occur in Dirac materials. By including explicit degrees of freedom for nuclear, electronic, and photonic interactions within the quasistatic approximation of the time-dependent density functional theory (TDDFT), we unravel how the intensity of the applied electromagnetic field distorts the underlying potential energy landscape of the Born-Oppenheimer hypersurface. We obtain topological phase diagrams to reveal distinct topological quantum phase transition (TQPT) points and study the signatures of quantum electrodynamics at these points. We demonstrate the formation of optical non-linearities in the topologically protected quantum phases and show that these propagate dynamically with increasing intensity of the symmetry-breaking field as standing waves modes.

We unravel unique TQPT points in the topological phase diagram as a function of the external drive field and show that the intrinsic SOC-induced semiconducting band gap in bulk TaAs is both scalable and field-tunable to obtain topologically ordered transport phases. The emergent transport phases considered herein are practically realizable in stacked multilayer heterostructure platforms that incorporate Dirac materials in monolayer or 2D form. The dependence of the magnitude of the emergent band gap on field tuning parameters shows that the carrier transport phase can be tuned from the trivial bulk semiconducting state to the topological insulating phase. Our study provides a unique framework for the rational understanding of the conversion of trivial equilibrium bands to non-equilibrium topological phases from engineered Floquet bands [16,17,18,19,20,21], photovoltaic Hall effect [22], photo-induced superconductivity [23,24,25], and the recently observed light-induced anomalous Hall effect in graphene [26]. Our results show that a switch between trivial and topological quantum states is realizable through the application of a perpendicular electric or magnetic field alongside a staggered-sublattice potential in the underlying lattice. Signatures of the near field electrodynamics show the formation of a quantum fluid-like phase at the topological quantum phase transition points. We discuss the implications of the field-induced carrier density wave phase and assert that the transmission of topologically protected carriers over this collective excitation mode is a possibility in optoelectronic and photonic applications.

This paper is organized as follows. In Section 2, details of the theoretical and computational methods are presented. These include first-principles calculations of the ground state in bulk TaAs, and its renormalization on the honeycomb lattice within an effective tight-binding model, to allow for the determination of emergent topological properties on the graphene lattice. In addition, the resulting near-field electrodynamics is computed in the quasistatic limit of the finite difference time-dependent density functional theory. In Section 3, the electronic structure and the derived topological phase diagrams are presented as a function of drive fields. The combined effect of a magnetic field, intrinsic SOC, and Zeeman spin-splitting fields on the topological stability of topologically ordered electronic phases are explored as a function of drive intensity, and the resulting optoelectronic and transport properties are analyzed in terms of the induced fields, transition contribution matrix, the photoabsorption, and carrier transmission spectra. Finally, conclusions are drawn in Section 4.

## 2. Theoretical and Computational Details

### 2.1. First-Principles Calculations

Tantalum arsenide (TaAs) crystallizes in a body-centered tetragonal structure with a non-symmorphic space group I41 md (No. 109) with lattice constants *a* = *b* = 3.37 Å, *c* = 11.56 Å [27]. The crystal structure lacks spatial inversion symmetry because there are two Ta atoms and two As atoms in each primitive unit cell, wherein each pair of Ta and As atom types are crystallographically non-equivalent. Firstly, collinear magnetization calculations were performed without spin-orbit coupling (SOC) for the structure relaxation using the plane-wave basis set, as implemented in the PWSCF code of the QUANTUM ESPRESSO suite [28,29]. Electron-ion interactions were described using PAW potentials [30]. Exchange-correlation energy correction as described in the generalized gradient approximation (GGA) as parameterized by Perdew, Burke and Ernzerhoff (PBE) [31]. Cut-off limits of 45 and 270 Ry we set for the kinetic energy and charge density expansions in the plane wave basis. The calculations were performed on a uniform Monkhorst-Pack k-point mesh of 8 × 8 × 8 (≈512) points, which is enough to achieve convergence of electronic energies and Hellman-Feynman forces to within 10^−12^ eV and 10^−3^ eV/Å, respectively. The Brillouin zone was sampled with a much denser *k*-points grid of 24 × 24 × 24 [32].

To impose time-reversal symmetry (TRS) constraints, electron states were treated as spinors with double group symmetry and populated using a Methfessels-Paxton smearing scheme with a smearing width of 7.35 mRy [33] since spin is a proper quantum number in Dirac materials. This constraint ensures that the electronic structure converged to the correct non-magnetic ground state. The lattice constants and internal coordinates of TaAs were fully optimized. Secondly, the relaxed structures were used as the input charge density for the calculation of the non-collinear magnetic ground state. In the non-collinear magnetization calculation, fully relativistic pseudopotentials were used. These inherently include the relativistic SOC as a first-order correction to the ground state electronic structure. SOC lifts Kramers’ degeneracy by splitting degenerate electron states. Using the DFT-level ground state, we compute the near field electrodynamics to unravel the optoelectronic and carrier transport response of dimers, small clusters, and bulk structures of TaAs and graphene in Section 3.

In Section 2.2, the ground state is renormalized to the graphene electronic structure and used to characterize the emergence of topologically ordered electron states. With the application of the external drive field to the rescaled graphene ground state, the response of the electronic structure to the applied external field is investigated. This scheme permits the analysis of the conversion of the trivial equilibrium bands into non-equilibrium topological bands as a function of the applied field intensity. In this approach, all the energy values used in the numerical computation are scaled by the hopping parameter *t*. This is expectedly different for chemically distinct 2D lattices like silicene, germanene, stanine, etc., with different *t* scaling compared to graphene.

### 2.2. Emergence of Topological Order in Electronic Phases

The simplest tight-binding model of the honeycomb lattice representation of a Dirac material is adopted to describe graphene with an orbital per site. The effective Hamiltonian of graphene is written in our tight-binding model as a sum of five contributions:

(1) $$H=H^{\mathrm{hc}}+H^{\mathrm{SO}}+H^{R}+H^{\mathrm{ST}}+H^{M}\;.$$

The terms denote contributions from the nearest-neighbor hopping, intrinsic spin-orbit, Rashba spin-orbit, staggered sublattice and magnetic exchange interactions. Therefore, the electrodynamics of a Bloch electron propagating in a 2D Dirac material under the effects of a uniform drive field [34,35,36,37,38] can be understood from the equivalent tight-binding formalism [37] of graphene. For the honeycomb lattice, this reduces to an effective Hamiltonian:

(2) $$H=-t\underset{\langle i,j\rangle \alpha }{\sum }{\;c}_{i\alpha }^{†}c_{j\alpha }\;\;+i\frac{\lambda_{\mathrm{SO}}}{3\sqrt{3}}\;\underset{\;\ll i,j\gg \alpha \beta }{\sum }\nu_{ij\;}c_{i\alpha }^{†}\sigma_{\alpha \beta }^{Z}c_{j\beta }\;+i{\;\lambda }_{R}\left(E_{Z}\right)\underset{\langle i,j\rangle \alpha \beta }{\sum }c_{i\alpha }^{†}{\left(\sigma \times {\hat{d}}_{ij}\right)}_{\alpha \beta }^{Z}c_{j\beta }\;-l\underset{i\alpha }{\sum }\mu_{i\;}E_{Z}c_{i\alpha }^{†}c_{i\alpha }+M\underset{i\alpha }{\sum }c_{i\alpha }^{†}\sigma^{Z}c_{i\alpha }\;\;$$

where $c_{i\alpha }^{†}$ ($c_{i\alpha }$) denotes the operator that creates (annihilates) an electron with spin polarization α at site *i*, and the sums over $\langle i,j\rangle$ and ≪i,j≫ run over all the nearest or next nearest neighbor hopping sites. The first term is the nearest neighbor (NN) hopping with unit energy *t*, which takes the value t≃ 2.7 eV [39]. The second term represents the effective of intrinsic spin-orbital coupling $\lambda_{\mathrm{SO}}$, where $\sigma =\left(\sigma_{x\;},\;\sigma_{y\;},\;\sigma_{z}\right)$ is the Pauli matrix of the spin, with $\nu_{ij\;}=+1$ if the next-nearest-neighboring hopping is anticlockwise and $\nu_{ij\;}=-1$ if it is clockwise to the positive *z*-axis. The third term represents the Rashba SOC $\left(\lambda_{R}\right)$ associated with nearest neighbor hoppings induced by external electric field *E_z_* [40,41,42]. The fourth term denotes the staggered sublattice potential, which is induced by the electric field *E_z_* and *l* denotes the buckle height of the lattice, where $\mu_{i\;}=\pm 1$. The last term represents the exchange field, *M* [40,43,44]. It is related to the magnetic field strength. It is equivalent to the total magnetic flux per unit cell $\Phi =\frac{1}{2\pi }\frac{gB_{0}}{\lambda_{z}}\mu_{B}$, where the Zeeman spin-splitting magnetic field effect on the spin-space Hamiltonian $H_{Z}$ is an on-site term. The exchange field *M* arises from proximity effect due to the coupling of the graphene sheet to a ferromagnet. This is obtained in multilayers that integrate a ferromagnetic slab in stacked heterostructures [10,11,12] or when ferromagnetic atoms are deposited on the graphene. The vector operation $\sigma \times {\hat{d}}_{ij}$ yields a spin matrix of zero diagonal and non-zero off-diagonal elements, such that hopping from site *i* to *j* leads to the flipped-spin configuration.

The emergence of topological order in the quantum transport phase of artificial-stacked multilayer systems plays an important role in modern condensed matter physics. However, detecting topological quantum phase transitions (TQPTs) is still a major challenge due to the absence of local order parameters. We address this challenge by tracking the emergence of topological order in the SOC-corrected DFT ground state of TaAs as a projection on graphene and tuning the TQPTs. By retaining the scaling parameters that reproduce the transport properties of the SOC-corrected TaAs band structure on the graphene lattice, we reproduce the correct low energy dispersion with *t* = 2.05 eV. Since other parameters like $\lambda_{\mathrm{SO}}$ and $E_{Z}$ are expressed in terms of this *t*, the emergence of topological order from the renormalized ground state and any renormalization to another buckled honeycomb structure depends explicitly on the ratios of $\lambda_{\mathrm{SO}}$ and $E_{Z}$ to the value *t =* 2.05 eV. The renormalized band structure is obtained with $\lambda_{R}/t=0.04\;t$ and *M*/*t =* 0.06 *t*. This guarantees transferability of *t* since the above scaling self-consistently adjusts to the competing internal fields to graphene. Our implementation uses QUANTUM HONEYCOMP version 0.19.1 (Jose Lado, Galicia, Spain). This is an open-source PYTHON utility for computing the topological, magnetic, and transport properties of quantum materials in the tight-binding approximation [45].

In the quantum Hall regime considered here, wherein quantized conductance has the notion of topological order intrinsically linked to the total flux Φ that passes through the Brillouin zone during gauge-invariant adiabatic cycling, distinct topological properties are known to emerge [46]. Quantized Hall conductance is thus the number of electrons transported across the Brillouin zone when Φ is increased by one unit of the flux quantum. As the graphene lattice is periodic in both *x* and *y* directions, both the vector potential A(*t*) and the effective Hamiltonian *H*(*t*) are also periodic in *x* and *y* directions, with the expectation value of the Hamiltonian *H* matrix obtainable in the Bloch functions basis via the discrete Harper eigenvalue problem [47]. Thus, the internal fields of the nonmagnetic bulk TaAs wherein the quantum state at 0 K are partitioned into an effective space and an orthogonal space and mapped onto the above effective Hamiltonian are adjusted to recover the unique spectral features in their low-energy band dispersion of graphene. With orbital hopping and self-energies described within a tight-binding model for nearest neighbor hopping on the hexagonal lattice, the two-band model of the dynamical bulk band structure was determined for the non-degenerate spin system subject to periodic boundary conditions.

The best candidate materials to realize TQPTs using applied external fields would be 2D materials with SOC. Since their monolayer form makes them suitable for integration into multilayer heterostructures coupled together through van der Waals forces, understanding their optical properties and characterizing how they respond to electromagnetic fields is crucial for optical and optoelectronic applications. We have implemented a combination of Rashba spin-orbit coupling effect, magnetic exchange field, and external electric field to drive a topological change in the quantum phase, which is tractable using the Chern number C and the $Z_{2}$ topological index. For simplicity, we consider a 2D crystalline system whose Berry connection of the *m*th band is:

(3) $$A^{\left(m\right)}\left(k\right)=i\langle u_{m}\left(k\right)|\Delta_{k}u_{m}\left(k\right)\rangle ,\;k=\left(k_{x},k_{y}\right)\;$$

so that the Berry curvature is given by:

(4) $$\Omega^{\left(m\right)}\left(k\right)=\Delta_{k}\times i\langle u_{m}\left(k\right)|\Delta_{k}u_{m}\left(k\right)\rangle$$

Thus, the Chern number of *m*th band is obtained as:

(5) $$C^{\left(m\right)}=\frac{1}{2\pi }\int_{\mathrm{BZ}}\Omega^{\left(m\right)}\left(k\right)dk,$$

where the integration is over the Brillouin zone (BZ). The Chern number is an intrinsic property of the band structure and has various effects on the carrier transport of the system [48,49]. In the presence of SOC, the effective Hamiltonian is analogous to the graphene quantum spin Hall effect (QSHE) Hamiltonian [50]. Thus, spin is a good quantum number in this formalism. Spin up and down Chern numbers individually serve as good topological invariants. Since spin (S_Z_) is a good quantum number in this two-band model of graphene, the $Z_{2}$ index is identical to the spin-Chern number $C_{S}$. These are defined when the topological state is gapped and the Fermi level lies within the gap region and given by: $C=C_{+}+C_{-}$ and $C=\frac{1}{2}\left(C_{+}+C_{-}\right)$, where $C_{\pm }$ is the summation of the Berry curvature in momentum space over the occupied electron states with $S_{Z}=\pm 1$. These metrics are well-defined even in systems where spin S_z_ is not a good quantum number [42,44,51].

### 2.3. Relationships with Other Models of Transport via Emergent Topological Quantum Phases

Over three decades ago, Schluter and Hybertsen [52] and Hybertsen, et al. [53] used two successive stages of the renormalization strategy to derive strong-coupling models for the electronic structure of La_2_CuO_4_ from results of local-density-functional calculations. In the first stage, they derived a 3-band Hubbard model with parameters calculated explicitly from first principles using a constrained density-functional approach and a mean-field fit to the Cu-O *pd*σ bands. In the second stage, they performed exact diagonalization studies of finite clusters within the 3-band Hubbard model to select and map the low-energy transport onto an effective one-band Hamiltonian, such as the Heisenberg, one-band Hubbard, or ‘*t*-*t*’-*J*’ model. They found that at each of the stages, the calculated observables were in quantitative agreement with experiments. They also observed that the insulating phase of La_2_CuO_4_ is quantitatively described by a Heisenberg model with excitation energies in good agreement with the experiment. Spectra for systems that have extra electrons or holes added were also found to be described well by the symmetric one-band models, which they suggested could form the basis for describing the superconducting transport state of La_2_CuO_4_. The second stage of the above renormalization approach, also known as the *t-J* formalism, has been used within the *d*-*p* model of the cell-perturbation method to describe CuO_2_ planes in cuprate superconductors [54].

Herein, we have applied the same renormalization strategies by considering both TaAs and graphene explicitly at two different levels of theory. At the first level, the electronic structure of TaAs is computed in its native 3D body-center cubic crystal structure using DFT. At the second level, the transport character that emerges from the SOC-corrected band structure of TaAs is considered based on the equivalent transport character of the 2-band tight binding level model of the field-tuned graphene model. This is an important aspect of the renormalization because the validity of the emergent quantum phases rests based on the accuracy of the DFT-calculated band structure of TaAs. Insights derived from results of the second level of theory constitute the theoretical basis for capturing emergent topologically ordered phases for quantum transport on the honeycomb lattice of the graphene.

The outcome of our renormalization is equivalent to considerations of the Kane-Mele model [55,56] for a bulk system, with an additional exchange field term and then fit the parameters such that the bandgap in the Kane-Mele model matches the bandgap in the DFT-computed TaAs band structure with SOC. Because the low-energy excitations of a Dirac material are uniquely described by the relativistic Dirac or Weyl equations [57], we have utilized TaAs and graphene as model lattice systems to describe the emergent carrier transport properties of a prototypical Dirac material. This permits the above modeling strategies, their results, and conclusions to become equally applicable to any Dirac material. However, we emphasize that the magnitude of each of the scaling parameters for electronic energy (i.e., *t*, *µ*, and *λ*) will differ as the platform used for the characterization of carrier transport is changed from one Dirac material to another.

The mapping that permits a generalization of our analyses to all Dirac materials is the rescaling of carrier transport from the low-energy band dispersion. This is so because the nature of carrier transport in any Dirac material (i.e., metallic, semi-metallic, half-metallic, semiconducting, or insulating, etc.) will depend strictly on the nature of the low-energy band dispersion around the Fermi level. Moreover, the limit of applicability of the results also extends to zero-buckled honeycomb structures, such as graphene and monolayer hexagonal boron nitride when integrated into stacked heterostructures insofar as the Hamiltonian parameters *t*, *µ*, and *λ*, etc. are rescaled to the correct DFT ground state in bulk TaAs. The validity of the mapping is dependent on the bulk-edge correspondence [8], which guarantees the same quantized conductance in any other spin-orbit coupled Dirac material at the correct set of field-tuning parameters.

More recently, Saxena, et al. [58] have used a similar set of rescaled energy parameters to study the effect of uniform disorder on the topological phase transitions induced by circularly polarized light in low-buckled spin-orbit coupled materials, such as silicene, stanene, germanene, etc. It is important to note that even though their model Hamiltonian is designed for characterizing materials with an intrinsically low-buckled hexagonal lattice structure, it is used to describe the carrier transport and topological properties that emerge from the edge states of the graphene nanoribbon. This approach has allowed them to identify a phenomenological *A*-phase, which appears in their topological phase diagram with the pair of spin-resolved Chern numbers (*C^↑^*, *C^↓^*) = (0,0) as the Floquet topological Anderson insulator phase. Their model showed that guaranteed topological protection of phenomena such as the sum-ruled quantum Hall conductance forms the basis for identifying phases and that these could serve as the signatures required to identify the individual phases in the topological phase diagrams. This is because, with the electronic signatures of edge states of the graphene ribbon, it is possible to circumvent the need to know the actual occupancy of bands involved in the transport.

To understand the efficacy and utility of our model to understand the charge or spin carrier dynamics, it is important to consider that when the transport platform, e.g., vertically stacked heterostructure system [10,11,12,13], is prepared such that carriers are transported in the steady-state, then the edge-modes of the incorporated Dirac materials can be made to acquire unit occupation with quantized conductance. The spin signature of such edge states is the crucial requirement for identifying unique phases in the calculated phase diagrams without using the occupancy of the band because spin is odd under time-reversal. The mapping that permits the analyses performed herein is the rescaling of carrier transport from the low-energy band dispersion. The validity of the mapping is dependent on the bulk-edge correspondence [8], which guarantees the same quantized conductance in any other spin-orbit coupled Dirac material at the correct set of field-tuning parameters. Srivastav, et al. [59] showed in thermal conductance measurements on graphene that the conductance quantization is a universal phenomenon that applies also to thermal transport on graphene and utilized the unique edge-state profile in graphene edge to obtain information on the topological order of heat carrier states.

Practically, spin carrier transport platforms in which the required broken TRS is provided by spin-sensitive and magnetic heterobilayer interfaces require an applied field to couple the spins to maintain time-reversal invariance. We show in Section 3.1.2 that under a suitable combination of internal (i.e., $\mu ,\;\lambda_{R,\;}\;\lambda_{SO}$) and external (i.e., *E*_Z_, *M*) fields, unique quantum transport phases emerge. In addition, markers of the local response of spin carriers are inferred from tuned graphene bands via the pair of topological invariants and Chern numbers, which quantify the real space topological order. Phases with order parameter pair (Z_2_,*C*) = (0,0) and (Z_2_,*C*) = (0,2) are equivalent to the band (i.e., trivial) insulator and topological insulator (TI) phases, respectively. Our results reveal that the magnitude of the inherently wide bandgap of this TI phase is scalable. In Section 3.2, we demonstrate further that the scalability of this bandgap and its field-dependent tuning leads to the emergence of an exotic quantum fluid phase, which we attribute to the topologically protected charge density wave transport state.

### 2.4. Time-Propagation TDDFT of the Topological Electronic Phase

Physical observables at the TQPT points depend on the response function of the underlying density matrix to the applied field. From the perspective of classical electrodynamics, it is intuitive to interpret the response of a material to light either as the absorption or scattering of the light, and such response is described by using Maxwell equations. However, one of the widely used numerical methods for obtaining computational solutions to Maxwell equations is based on the finite-difference time-domain (FDTD) approach [60]. The FDTD approach is based on the time propagation of the electric and magnetic field components of the applied electromagnetic radiation in a way that allows observables of the field-induced response to be expressed on real space grid points. Optical constants are derived from the resulting far-field pattern. In the microscopic limit where short length and time scales dominate, the quasistatic approximation of the FDTD approach (QS-FDTD) is valid. Computational implementation of the QS-FDTD approximation allows the retardation effects of the finite speed of light to be neglected insofar as the length scales are small, typically below ~50 nm, for DFT calculations of the electronic structure within the supercell approximation to remain valid. Compared to full FDTD, the quasistatic formulation has some advantageous features. The magnetic field is negligible and only the longitudinal electric field needs to be considered so that the number of degrees of freedom is smaller. Because the retardation effects and propagating solutions are excluded, longer time steps and simpler treatment of the boundary conditions can be used. The approximation allows for the derivation of an alternative set of time-dependent equations for the polarization charge, current, the electric and magnetic fields.

By treating the electronic structure as an autonomous quantum system such that its Hamiltonian is dependent on time, then all relevant information about the system is contained in the matrix elements of its time evolution operator. As such, the reduced matrix elements of the single-particle density are recoverable from frequency space Fourier transform of the ground state density matrix since it constitutes the time propagator of the system within QS-FDTD. The time propagator gives the probability amplitude for the electron state to propagate between Point 1 (denoted by wave vector *k*_1_) at time *t*_1_ and Point 2 (denoted wave vector *k*_2_) at time *t*_2_ due to perturbation of the dipole moment within the interval of time Δ*t* = *t*_2_ − *t*_1_. This is equivalent to the application of non-zero field to the single-particle ground state density at time interval Δ*t*. The system propagator gives the probability amplitude for the electron state to propagate between Point 1 (wave vector *k*_1_) at time *t*_1_ and Point 2 (wave vector *k*_2_) at time *t*_2_ in reciprocal space due to the perturbation of the dipole moment within the interval Δ*t*. This is equivalent to the application of zero and non-zero radiation fields to the single-particle ground state density at time *t*_1_ and *t*_2_, respectively. For instance, in their microscopic theory of the field-dependent carrier dynamics, Sato et al. have treated the time evolution of the reduced density matrix *ρ* under a phenomenological relaxation *D* using a quantum Liouville equation [61].

Hereunder, the time-propagation TDDFT approach of describing electronic states in the presence of an applied field [62] is adopted to compute the time-dependent density matrix (i.e., propagator) of the system $n\left(r,t\right)=\underset{\mathrm{occ}}{\sum }\left|\Psi {\left(r,t\right)}^{*}\Psi \left(r,t\right)\right|\tilde{r}$ using the corresponding time-independent all-electron wave function $\Psi \left(r\right)$ in the basis set of atomic-like functions. In the projector augmented wave (PAW) formalism, the time-dependent Kohn-Sham equation is represented using the PAW projector $\hat{T}$ of the all-electron wave function $\Psi \left(r,t\right)=\hat{T}\;\tilde{\Psi }\left(\tilde{r},t\right)$ as:

(6) $$\left[{\hat{T}}^{†}\left(-i\frac{d}{dt}+{\hat{H}}_{\mathrm{KS}}\right)\hat{T}\right]\Psi \left(\tilde{r},t\right)=0$$

The corresponding time-dependent wave functions $\tilde{\Psi }\left(r,t\right)$ are represented using a basis constructed from the linear combination of atomic-like orbital (LCAO) functions $\tilde{\phi }\left(r-R\right)$, which are centered on atom μ located at point *R* as a discrete sum over all atoms as,

(7) $$\tilde{\Psi }\left(r,t\right)=\underset{\mu }{\sum }\tilde{\phi }\left(r-R_{\mu }\right)c_{\mu n},$$

such that the matrix elements of the time-evolution operator are derived from the coefficients of the LCAO wave function as:

(8) $$iS\frac{d}{dt}C\left(t\right)=H\left(t\right)C\left(t\right).$$

The terms **C**, **S**, and **H** denote dense matrices of linear Hermitian operators. The numerical implementation relies on the semi-implicit Crank–Nicolson method to propagate the wave functions in time. For a given wave function $C\left(t\right)$ at time *t*, the system is propagated forward by using the time-dependent Hamiltonian **H**(*t*) to solve the linear equation for the predicted matrix elements of the wave function $C^{\prime }$ at the forward time $\left(t+\Delta t\right)$, while $H\left(t\right)$ is computed at the midpoint of the time-step δt according to the condition:

(9) $$S+iH\left(t\right)\frac{\delta t}{2}C^{\prime }\left(t+\delta t\right)=S-iH\left(t\right)\frac{\delta t}{2}C\left(t\right).$$

With the predicted wave function $C^{\prime }\left(t+\delta t\right)$, the time-updated Hamiltonian $H^{\prime }\left(t+\delta t\right)$ is computed at the midpoint of δt as:

(10) $$H^{\prime }\left(t+\delta t\right)=\frac{1}{2}\left[H\left(t\right)+H^{\prime }\left(t+\delta t\right)\right].$$

By using the corrected Hamiltonian $H^{\prime }\left(t+\delta t\right)$, the system is propagated further in time *t* + δ*t* by computing the updated wave function $C^{\prime }\left(t+\delta t\right)$ as a numerical solution to the

(11) $$S+iH\left(t+\frac{\delta t}{2}\right)\frac{\delta t}{2}C\left(t+\delta t\right)=S-iH\left(t+\frac{\delta t}{2}\right)\frac{\delta t}{2}C\left(t\right).$$

Diagonalization of matrices **C**, **S**, and **H** are handled with ScaLAPAC and BLACS for the QS-FDTD computations within the grid-based projector augmented wave code, GPAW [63]. The above numerical procedure was implemented over a pulse duration Δ*t* of 40 fs over a total of 2000 simulation steps *N* using a timestep δt of 4 attoseconds (4 × 10^−18^ s), where Δt=Nδt. The SICN algorithm has an embedded Euler step in each predictor step. This makes the time propagation an efficient numerical operation over the *N* simulation steps:

(12) $${\tilde{\Psi }}_{N}\left(t+\delta t\right)\approx \left(1-i\;{\hat{S}}_{approx.}^{-1}\left(t\right)\hat{H}\left(t\right)\delta t\right){\tilde{\Psi }}_{N}\left(t\right),$$

where the matrix operation ${\hat{S}}_{approx.}^{-1}$ denotes an approximate inversion of the overlap matrix $\hat{S}$.

Because the external field effect can be complicated when the intensity is strong enough to distort the positive Coulomb potential of the underlying lattice, we only consider the response of the ground state density in the weak-field limit. In this case, the applied field is treated as an absorption kick through a weak delta-function pulse of the dipole electric field. By allowing the electronic structure to propagate freely, we also obtain the time average of the pseudo electron density $\bar{n}\left(r,t\right)$ on a set of *k*-grid points as a response over the propagation time $t_{N}$ as,

(13) $$\bar{n}\left(r,t\right)=\frac{1}{t_{N+1}}\left(\sum_{0}^{N}\left|\tilde{n\;}\left(r,t\right)\right|\Delta t\right).$$

In this case, setting the simulation time to satisfy the condition: $t_{N+1}=\overset{j=N-1}{\underset{j=1}{\sum }}\Delta t_{j}$ overall steps allow for the efficient computation of the time-dependent dipole moment, the absorption spectrum, and the induced electric fields from the time-averaged density matrix or propagator $\bar{n}\left(r,t\right)$. Since it is only the excitations that have been induced by the absorption kick that can show up in the absorption spectrum, we have also computed the Fourier transform of the density matrix on discrete grid points. This was obtained in the frequency domain, as a discrete moving-average Fourier transform of the pseudo-charge density $\tilde{n}\left(r,t\right)$ relative to the time-averaged density $\bar{n}\left(r,t\right)$ over each simulation step as:

(14) $$F_{N}\left(r,\omega \right)=\frac{1}{\sqrt{\pi }}\left(\sum_{0}^{N}\Delta n\left(r,t\right)\;e^{-\frac{1}{2}\;t_{N}^{2}}e^{i\omega t_{N}}\Delta t_{N}\right),$$

where $\Delta n\left(r,t\right)=\tilde{n}\left(r,t\right)-\bar{n}\left(r,t\right).$ The above algorithm leads to non-trivial numerical challenges with storage of large data that must be computed on arrival at time *t*_N_ on a large number *N* of real-space grid points, typically *N* = 10,000. This challenge is surmounted by implementing a numerical strategy that only requires data to be computed one timestep at a time.

## 3. Results and Discussion

### 3.1. Electronic Properties

#### 3.1.1. Renormalized Electronic Ground State

**Figure 1:**
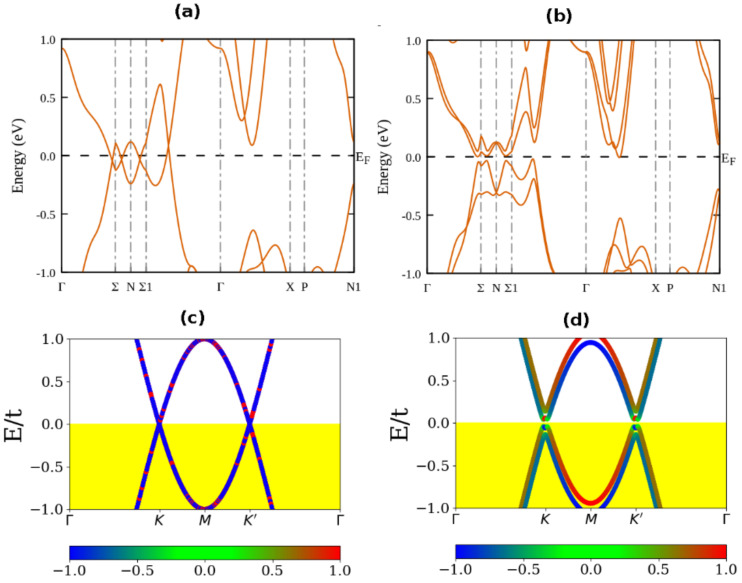
The DFT electronic structure of bulk TaAs in the absence of SOC (**a**). In the presence of SOC (**b**). Where the dashed horizontal line denotes the Fermi level. The corresponding band structure of bulk TaAs renormalized to the graphene sheet in the absence of SOC (**c**). In the presence of SOC (**d**) at $\lambda_{R}/t=0.04t$ and exchange field *M*/*t =* 0.06 *t*, with the Fermi level located at *E*/*t* = 0 eV, where *t* denotes the rescaled unit of energy. The color bars in (**c**) and (**d**) denote the value of the spin texture in units of *S*_Z_, where *ħ* =1.

Figure 1 shows the bulk band structures of TaAs and the graphene sheet as a benchmark of the renormalized electronic structure. In the absence of SOC, the conduction and valence bands of TaAs cross along the Ʃ-N-Ʃ_1_ direction of the Brillouin zone (Figure 1a), as expected from a semimetal. In the presence of SOC, by contrast, the band structure is fully gapped along with the high-symmetry directions (Figure 1b). In addition, the Weyl points that appear in the presence of SOC have shifted away from the high-symmetry points, and the double degeneracy of bands is lifted except at the Kramers points. This splitting of energies confirms the lifting of degenerate electron states due to the presence of intrinsic SOC. Our PBE calculations of the TaAs band structure agree with published band structures calculated for TaAs [64,65,66]. As a Weyl semimetal, Yan, et al. [67] described bulk TaAs as the 3D analog of graphene because of its linear dispersion around the Weyl points. 

The two-band tight-binding model of graphene sheet yields the zero-gap band structure in the absence of SOC (Figure 1c) and a gapped band structure in the presence of SOC (Figure 1d). This electronic structure is consistent with the semi-metallic transport expected in the pristine graphene sheet. The corresponding spin texture of the renormalized band structure is shown as the color bar. Although the value of the SOC in graphene is too small to open a sizeable band gap in graphene, it is important to emphasize that the role of the SOC is to lift the Kramers degeneracies that occur in electron band states when SOC is ignored. Nevertheless, the presence of intrinsic SOC in a Dirac material does not break the time-reversal symmetry of the electronic structure because the Hamiltonian and the SOC-operator commute. The invariance of the SOC under time reversal symmetry means that additional fields that can break the symmetry of the electronic structure must be applied to the electronic states to open the bad gap in bulk TaAs. These additional fields are phenomenological. In the actual calculations for bulk Dirac materials, these fields are modelled as the Rashba SOC $(\lambda_{R}),$ the magnetic exchange (or Zeeman) field M and the external electric field *E_Z_*. Both the SOC-corrected band structure of bulk TaAs and the renormalized graphene electronic structure are semi-metallic as expected. The SOC effect is weak in graphene compared with other 2D materials such as silicene or germanene. Nevertheless, its low-buckled crystal structure introduces a low-staggering potential, with important ramifications on the dynamical electronic structure.

By contrast, in material platforms wherein a surface slab of the Dirac material (e.g., TaAs) has been combined with a magnetic layer to form a bilayer or a multilayer heterostructure with perpendicular magnetization the scenario is different. In this case, both structural inversion and time-reversal symmetries are broken. The broken symmetries introduce edge as well as interfacial states into the electronic structure at ground state. Thus, without any additional fields, the hybridization, or electronic exchange coupling, between the surface or edge states and the magnetic ordering in the magnetic layer are conditions that could open a band gap at the Dirac point. When the heterostructure also incorporates a topological insulator within the stack, then either the broken time-reversal symmetry or spatial inversion symmetry of a Dirac point can lead to the appearance of Weyl points.

We emphasize that the renormalization of the ground state is effective in the regime wherein the driving field intensity is sufficiently weak for its effects to be included through a time-dependent vector potential without altering the underlying lattice system. In this approach, experimental results for conversion of trivial equilibrium bands into topological non-equilibrium bands in both low [16,17,18,19,20,21] and high [68] frequency limits are understood in terms of non-stroboscopic and stroboscopic electron dynamics. We also demonstrate how the external drive can be used to modify the trivial band structure as a necessary and sufficient test of the renormalization strategy. In Section 3.2, we demonstrate how the propagator of the system is recoverable from the topological phase diagram by considering the recovery in the two physically relevant limits in which there are zero and non-zero radiation effects on the ground state.

Figure 1d shows that the application of Rashba SOC of $\lambda_{R}/t=0.04t$ and the Zeeman exchange of *M*/t = 0.06 t opens a small band gap in the graphene. The size of the band gap in graphene is equivalent to the SOC-induced band gap in TaAs. The fact that the magnitude of this induced band gap exactly matches the SOC-induced gap in TaAs (see Figure 1b) is the underpinning basis for the ground state renormalization. In addition to the Weyl and Dirac nodes, there are several other possibilities for the formation of the zero-band gap electronic structure. Figure 1a,c shows the electronic band structure when time-reversal and spatial inversion-symmetries are preserved at generic points in the k-space that are not necessarily high-symmetry points. The drive field lifts the degeneracy at the high symmetry points where the valence and conduction bands touch. As expected, the ground state band structure shows semi-metallic transport character in the absence of SOC since the bands at Brillouin zone point K and K’ touch each other, with an equivalently small bandgap opened in the DFT band structure of TaAs (see Figure 1b,d). These show that the SOC-corrected TaAs band structure is characterized by a small bandgap. The SOC also lifts the degeneracy of electron states, and this assigns extremal spin textures, i.e., S_Z_ = ±1 to the two bands that lie within both conduction and valence bands at the M-point of the Brillouin zone. Thus, it is not the band structure of the graphene itself that captures the sensitivity to spin. This is because the size of the intrinsic SOC in graphene is very small [69]. It is instead the SOC-induced spectral gaps at high-symmetry points [70] that respond to the field.

From Figure 1a, it is observed that two of the four TaAs bands that cross the Fermi level at the midpoint along the Σ-*N* and *N*-Σ_1_ directions of the TaAs Brillouin zone (BZ) denote the two bands modeled by the graphene band crossings at points *K* and *K*’ in Figure 1c. This is because the magnitude of the SOC-induced band gaps at these two pairs of BZ points is both minimal and equal (Figure 1b). As such, these limit the overall transport character of the system to the semiconducting state notwithstanding the magnitude of any other gap determined at any other BZ point and the differences in their local structure and chemistry. Thus, the carrier transport character that results from the SOC-corrected TaAs band structure around the Fermi level is correctly approximated by the emergent transport character of the *tuned* graphene, as modeled in the 2-band tight-binding approximation at a given set of field-tuning parameters. This specific set of field-tuning parameters for TaAs gives unique carrier transport signatures on the honeycomb lattice. As such, a one-to-one correspondence exists between the carrier transport character of the 3D body-center cubic TaAs as a mapping to the equivalent transport character of the field-tuned hexagonal lattice of graphene.

The above renormalization strategy is valid insofar as the mapping is not construed to imply that any form of physical transformation of the 3D body center cubic TaAs lattice structure into an equivalent 2D hexagonal lattice representation exists for the same material. This is because no physically intuitive information on carrier transport is lost in this process. The electronic structure presented herein is neither a mapping to establish an equivalence between the underlying lattices in TaAs and graphene, nor to establish the equivalence between their essential chemistries. Instead, it establishes that the emergent carrier transport character in the electronic structure of TaAs around the Fermi level after the SOC-correction is equivalent to the suitably tuned graphene electronic structure. Consider that TaAs is ordinarily semi-metallic in the absence of the SOC, which upon field-tuning yields an equivalent semiconducting character just as graphene. This emergent carrier transport matches the semiconducting behavior observed in the band dispersion along the Γ-Σ-N-Σ_1_-Γ direction of the BZ in TaAs, notwithstanding the actual point in the BZ at which the carrier transport limiting bandgap, is opened. The one-to-one correspondence established in the above renormalization procedure is used in the following to track the emergence of topologically ordered quantum transport phases from the field-free semiconducting transport phase of TaAs (Figure 1b). This is performed in a representation in which the SOC-corrected DFT ground state of TaAs has been mapped onto an equivalently gapped graphene band structure where the emergence of topological order is captured.

#### 3.1.2. Characterizing the Topological Order and Quantum Phase Transitions

The use of topological phase diagrams to identify electronic phases is presented below. By analyzing the low energy dispersion of electronic phases, we demonstrate the emergence of non-local topological order and identify the associated TQPTs using topological order parameters. To check that the BEC principle correctly preserves the chiral edge states expected on the graphene ribbon, the corresponding band structures are shown for special Brillouin zone points in each topological phase and labeled with quantized topological order parameters. Topological invariant $\left(Z_{2}\right)$ and Chern number (*C*) is used to characterized phases for time-reversal invariant electronic systems, where $Z_{2}=1$ denotes a non-trivial electronic phase such as the topological insulator phase (TI) or the quantum spin Hall insulator (QSH) phase, whereas $Z_{2}=0$ indicates trivial phase such as band insulator.

In the low-energy effective theory of the electrons, carrier dynamics is described by the Hamiltonian $h_{\pm ,0}\left(q\right)=E_{0}\left(q\right)\;\sigma_{0}+V_{x}q_{1}\;\sigma_{x}+m_{0}\sigma_{z},$ where $q=K-K_{0}$, *σ* denotes the Pauli spin matrices and mass *m* is a tuning parameter for the TQPT [71]. The TQPT between the QSH and band insulator (BI) phases is distinguished only by the $Z_{2}$ index. This occurs only when the mass *m* term in the Hamiltonian $h_{\pm ,0}\left(q\right)$ changes its sign. The argument used to determine the change of $Z_{2}$-index allows for classification of phase transitions in terms of the changes between the expectation values of the order parameter of two distinct time-reversal invariant topological and trivial phases [72]. The presence of both time-reversal and inversion symmetries guarantees that order-parameter *C* vanishes. Close to the transition point between two quantum phases, the electronic structure is prone to strongly enhanced field-dependent responses, which are tractable with topological quantum numbers, $Z_{2}$ and C.

Topological order parameters that characterize the QSH and band insulator phases are often a pair of integers ($Z_{2},\;C$). The integral form of the order parameters ($Z_{2},\;C$) for an electronic phase denotes quantization of the topological order, and their discontinuous change signals a TQPT. For the applied drive intensity to spontaneously break the symmetry of the Hamiltonian of the electronic system as to warrant a TQPT, at least one of the parameters of its Hamiltonian must be tuned through a critical value. In addition, the resulting phase transition must signal a change from one state of quantum matter to another. This phase change could be from a trivial to a topological electronic phase and vice versa. Both $Z_{2}$ and C are proper quantum numbers that are necessary to characterize the state of quantum matter at 0 K as trivial or topological. These metrics indicate the global properties of the state manifold of the quantum matter defined on the irreducible Brillouin zone (IBZ). Because all quantum states that belong to the same topological sector of the IBZ are homotopic, they can be continuously deformed from one state to another or driven in-between states without closing the bulk energy gap by applying a symmetry-breaking field. Therefore, at the point of TQPT, the state manifold must experience a discontinuous change in configuration. The changes manifest as a sign-change in the mass term of the Hamiltonian can also show up through the inversion of electronic bands in TIs since the bandgap closure is guaranteed by the BEC principle as an effect of broken time-reversal symmetry.

#### 3.1.3. Tuning the Topological Order Using Material-Dependent Potentials

**Figure 2:**
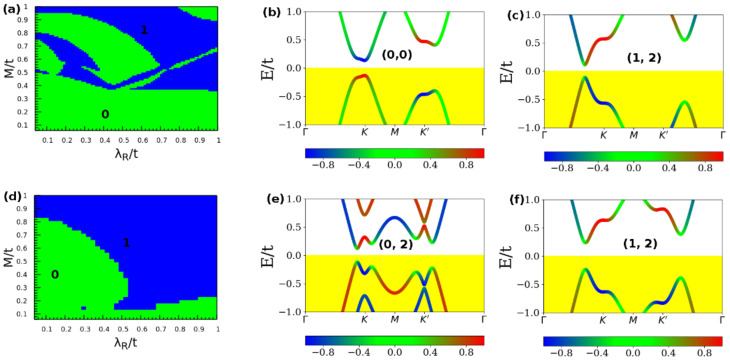
Effect of a fixed staggered sublattice potential of 0.1 *t* on the topological invariant phase diagram of the Haldane model on the modified honeycomb lattice (**a**). The associated band structure of identified electronic phases in phase fields is indicated by the blue color (**b**) and green (**c**). A similar plot at a fixed staggered sublattice potential of 0.3 *t* (**d**) and the band structures are associated with the two-phase regions denoted by color blue (**e**), and green (**f**). The color bars in (**b**,**c**) and (**e**,**f**) denote the expectation value of the spin texture in units of *S*_Z_, where *ħ* = 1.

In this section, the topological phase diagrams and the corresponding band structures derived from the applications of global symmetry-breaking fields are analyzed to demonstrate that the occurrence of the TQPT points are tunable through the application of fields-both intrinsic and extrinsic to the material Therefore, it is important to understand how the above characterization of topological order and the resulting TQPTs would change when the renormalized electronic structure of the TaAs ground state is projected instead onto any other 2D material with intrinsic honeycomb structure apart from graphene. To this extent, the effect of the staggered sublattice potential *µ* on the electronic structures is explored. This effect originates from the low buckled honeycomb structure. As such, *µ* is different in graphene and any other Dirac material for the tuned honeycomb model. Figure 2 shows the influence of the staggered sublattice potential on the topological invariant phase diagram of the Haldane model on the honeycomb lattice of graphene [73], and the derived band structure showing distinct TQPTs. The phase diagrams represent the response of the ground state to changes in the internal field due to the staggered potential *µ*. From the sensitivity of the topological order parameters and the resulting electronic phases to *µ*, we now demonstrate that a facile electronic switch is obtainable on these materials by suitably tailoring the internal fields. This way, the low energy transport character can be tuned from the low energy dispersion of its topological edge states, and thus tune the TQPT.

**Figure 3:**
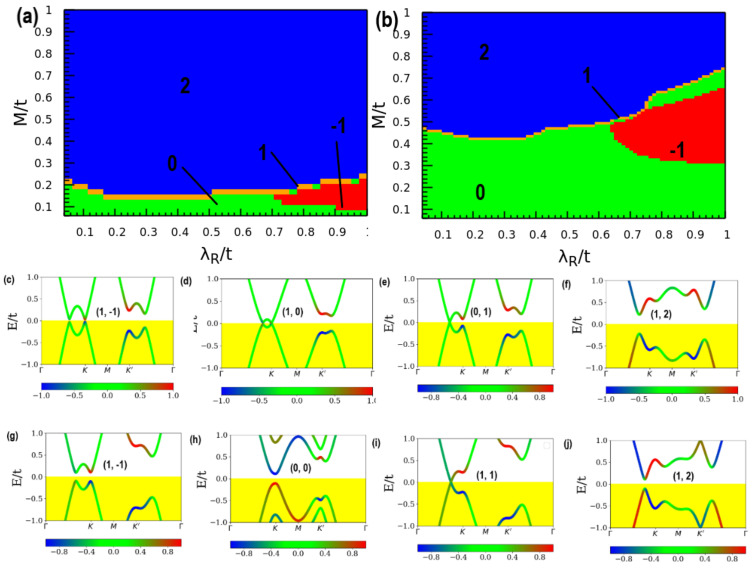
Chern number phase diagram as the function of staggered sublattice potential *µ*_1_ = 0.1 *t* (**a**). *µ*_2_ = 0.3 *t* (**b**). Where green denotes *C =* 0, blue denotes *C =* 2, red denotes *C* = −1 and yellow denotes *C =* 1, respectively. The band structure derived for unique quantum phases at 0.1 *t* are plotted for Chern numbers: −1 (**c**),0 (**d**), 1 (**e**), 2 (**f**). The corresponding band structure for unique quantum phases at 0.3 *t* are plotted for Chern numbers: −1 (**g**), 0 (**h**), 1 (**i**), 2 (**j**), respectively. The color bars in (**c**) to (**j**) denote the expectation value of the spin texture in units of *S*_Z_, where *ħ* = 1.

Figure 2a shows the topological invariant phase diagram of the Haldane model obtained on the modified graphene lattice with *µ*_1_ set to 0.1 *t*. This also exhibits a two-phase region identified with Z_2_ *=* 0 (green) and Z_2_ = 1 (blue). Figure 2b) shows the modified band structure of the (0,0) trivial electronic phase at 0.1 *t*. The band structure is characterized by a small bandgap at the *KK’* points of the IBZ and the blue, red, and green regions in the band structures (Figure 2b,c,e,f and 3c–j) denote the up spin (↑↑), the down spin (↓↓) and edge states respectively. Besides, the nature of the low energy dispersion is fundamentally different relative to the band structure of the equivalent electronic phases. In addition, *µ* has significantly influenced the low-energy dispersion but without altering the underlying topological order of the phase since the pair of order parameters (0,0) remains unchanged. Figure 2c shows the band structure of the electronic phase after the TQPT has occurred under the lattice distortion effect due to *µ*_1_. This shows that this TQPT has been accompanied by a considerable closure of the bandgap although the overall electronic structure is still gapped of the transport character of the quantum state is unchanged.

The effect of setting the staggered potential *µ*_2_ = 0.3 *t* in the topological invariant phase diagram of the Haldane model is shown in Figure 2d. In addition, the corresponding band structures are shown in (Figure 2e,f) it is thus concluded that the effect of *µ* on the band structure of this electronic phase is non-negligible–both from the perspective of carrier transport and the topological order. We have noted that only the two indicated values of the staggered potential can yield a transition between trivial and non-trivial quantum phases and vice versa. At any given staggered-potential, phase transition lines separate the trivial insulating (denoted by *C* = 0) and non-trivial insulating (denoted by *C* = +1) Chern phases. In Figure 2e, the Chern number changes by three units from ±1 to 2 across phase boundary lines due to the creation of the three satellite Dirac points at the *KK’* points. The appearance of non-zero Chern numbers (see Figure 2c,e,f) guarantees the existence of gapless edge states due to the topological index theorem [74]. To summarize, the Haldane model on the honeycomb lattice has four phases made up of three topological phases with *C* = +2 and one trivial insulator phase with *C* = 0.

Consider that the Chern number of a band can only change when it crosses another band at the TQPT point. The transport signature of the electronic phase must be either metallic or semi-metallic, i.e., without an insulating gap. On the contrary, *C* = 0 guarantees the existence of the trivial insulator phase in the Haldane model, we assign the opening of the *KK’*-point bandgap to the formation of the topological insulating phase from the trivial band insulator phase. In massive Dirac fermion systems, the BEC principle guarantees the existence of a chiral edge state per boundary of the system. Thus, when the Fermi level is located within the gap region, the case where *C* ≠ 0 means that bands must appear below the Fermi energy. This implies that both (integer) quantum Hall and gapless chiral edge states must coexist in the presence of disorder. For this reason, we suggest that such exotic quantum phases are realizable in artificially stacked van der Waals multilayer heterostructures [10,11,12,13] when engineered to incorporate layers of heavy metal species due to the presence of strong SOC and intrinsic long-range disorder.

We have also monitored the effects of changes in the intrinsic SOC term on the emergent electronic phases. The contribution is small in graphene and but can also be enhanced on purpose. For instance, time-reversal symmetry can be tuned through electrostatic doping of the ferromagnetic (FM) layer [75]. In artificially stacked multilayer heterostructures [10,11,12,13], this is achievable by incorporating a heavy metal (HM) layer with large intrinsic SOC. The use of the HM layer or electrostatic doping with an FM layer breaks time-reversal symmetry. This ensures that tunability of the inherent topological order of the ground state is guaranteed. By contrast, the introduction of sublattice asymmetry also breaks the inversion symmetry of the graphene layer to open a trivial bandgap. This also occurs in the ground state of hexagonal boron nitride monolayer [76,77]. The asymmetry is realizable through substitutional doping, lattice symmetry engineering, and the application of uniaxial or biaxial strain. It is important to emphasize that since the sublattice asymmetry leads to different hoppings terms in the effective Hamiltonian, the Dirac points are guaranteed to shift away from the *K* and *K’* points.

To illustrate the sensitivity of the electronic structure to the underlying material, we have also investigated the Chern number phase diagram at two different constant intensities of the staggered sublattice potential. Material dependence is modeled in terms of a fixed value of the staggered sublattice potential. To maintain consistency with the previous section, the staggered sublattice potential has been fixed. Figure 3 shows the Chern number phase diagram at staggered sublattice potential *µ*_1_ = 0.1 *t* (Figure 3a) and *µ*_2_ = 0.3 *t* (Figure 3b), and the band structure of unique quantum phases at the computed Chern numbers. We also find that changes in the Rashba SOC independently induces a finite bandgap in all the band structures shown in Figure 3. The corresponding topological order parameters result in a series of trivial-to-topological phase transitions as the parameters are ramped up suggesting that the intensity of the drive field plays a non-ignorable role in the TQPT, but once a transition the topological phase occurs, intensity changes do not have any further effect.

From Figure 3a,b, the Chern phases are highly sensitive to the small range of the changes in the Rashba SOC. For instance, not only are the areas of the non-trivial Chern phase greatly enlarged but also the values of Chern number are changed. This general feature is observed when the underlying transport character and SOC parameter of the effective Hamiltonian change under the drive. Figure 3c–f suggests that as the Kramers degeneracy of the bands is lifted at the *KK*′ point, it is substantially easier for Chern phases to form. A similar manner has also tuned the Z_2_ invariant as a function of Rashba $\lambda_{R}/t$ and exchange field (Zeeman) *M*/*t* with a fixed value of electric field *E_z_/t =* 0.5 *t*. This value of the applied field yields a TQPT and corresponds to an absorption kick on intensity 0.01. Nevertheless, it is shown in Section 3.2.1 that even lower intensity fields are enough to create non-zero components in the optical transition matrix. The transition from trivial to topological electronic phase is observed and the bandgap increases when the driving field intensity increased. When the Rashba field is increased, the bandgap is decreasing systematically, leading to gap closure near *K* and *K′.* This also demonstrates the ability to tune a normal insulator into a topological insulator using the electric field since the Chern number for both cases is *C* = 2.

Analyses of our results on the Haldane model (Figure 2) demonstrate that if the value of *C* is non-zero, all the topological phenomena expected in the quantum Hall transport state will be observable, including the quantized Hall conductivity and the existence of the edge states. In addition, Figure 3 illustrates the use of fixed staggered sublattice potential, as an internal field constraint of the transport platform, to also achieve the tunability of the SOC-induced semiconducting gap in TaAs. For instance, (Figure 3c,g,h,j) show the semiconducting band structure while Figure 3f shows the insulator band structure. On the other hand (Figure 3d,e,i) each shows the metallic band structure. These show that the carrier transport phase is scaled by the magnitude of the bandgap and tunable with *μ*. The range of the bandgap, which spans from zero (metallic), minimal (semiconducting) to maximal (insulating) indicates that the is scalable under the constraints of broken time-reversal symmetry. Our results show that both intrinsic ($\lambda_{\mathrm{SO}}$) and Rashba ($\lambda_{R}/t$) SOC are key parameters for obtaining quantized Hall conductivity under the fixed staggered sublattice potential *μ* in time-reversal symmetry invariant systems because there is no spontaneous magnetic moment. These imply that in both TaAs and graphene, the effect of an external magnetic field can play the role of the two forms of SOC contributions [55,56].

### 3.2. Optoelectronic Properties

Signatures of the near field quantum electrodynamics in the topological electron phase are analyzed below to reveal a quantum fluid-like phase as a collective excitation mode for carrier transport. This is central for understanding the non-linear optoelectronic response of Dirac materials at the TQPT point. The response of the underlying chemical bonds to light, and the light-induced interband transitions are strongly sensitive to the ground-state density and the intensity of the applied field. To understand these electronic responses, we also investigated the contributions from the optical transition matrix to the photon absorption spectrum due to the distinct peaks identified in the photoabsorption spectrum. Our results show that apart from the selective absorption of photons at critical fields wherein TQPT is found to occur in the two models, there are also significant increases in the intensity of the induced field around constituent atoms, even at very weak drives. The atomic site resolution of the intensity of the induced fields is presented and discussed to reveal wavelike quantum interference patterns as the field-dependent response of the underlying electronic structure to carrier transport.

#### 3.2.1. Near-Field Electrodynamics of Topological Electronic Phases

The theoretical models presented above are useful for understanding emergent carrier transport in bulk Dirac materials. However, their implementation on transport platforms derived from bulk Dirac material is computationally intensive. When a Dirac material is incorporated into the van der Waals multilayer stack, its surface and edge states support topologically protected states for tunable carrier transport. Quite fortunately, it is nanosized (not bulk) components of Dirac material that are integrated into heterostructures for device applications where oscillations of the free-electron density and spin sensitivity of heterobilayer interfaces are crucial. Such interfaces typically require the integration of 2D (or monolayer) material forms into vertical multilayer stacks or lateral heterostructures. Thus, the emergent quantum transport phases modeled herein are readily realizable using suitably engineered multilayer heterostructure platforms. Due to system size limitations, we have extended the computational implementation from periodic 3D bulk Dirac materials to their equivalent but smaller-sized 2D and 1D systems such as nanoclusters and atomic nano-line, as structural models of TaAs and graphene nanoparticles.

This computational approach allows us to gain insights into the plasmonic signatures that arise from optoelectronic responses of the free electron gas when nanoclusters and single-bond geometries of Dirac materials have been integrated into artificially stacked materials platforms. We emphasize that the presence of coupled heterobilayer interfaces in such coupled heterostructure introduces long-ranged structural disorder which guarantees the broken spatial inversion symmetry expected in bulk Dirac materials. Recently, the use of multilayer heterostructures that incorporate Dirac materials in 2D (or monolayer) form for spintronic memory applications has been demonstrated [78]. The calculations are tractable using TaAs and graphene models of Dirac materials because they retain their chiral edge states in nano nanocluster geometries. Expectedly, retention of the chiral edge states at reduced system size means that quantum confinement effects also become relevant. The transport signatures that arise from edge-modes will still be topologically protected. The tunability of the preserved topological order in reduced geometries is guaranteed by the spontaneous appearance of a pair of topological invariant numbers (Z_2_, *C*), as discussed in Section 3.1.2.

To obtain deeper insights into the fundamental physics of the interaction between the applied field and the ground state density in topologically protected electronic phases, we emphasize that time propagation of the ground state density is implemented based on real-time propagation of atomic-like basis functions and not plane waves. As such, the interacting density response function of a periodic structure cannot be described within periodic boundary conditions because periodically repeating unit cells are not suitable to describe the non-periodic structures investigated here. For clusters such as nanoparticles, nanolines, and small molecular fragments, it is not necessary to define an upper bound for the electrostatic potential for the field-structure interaction. Instead, it is only necessary to ensure that the reference energy for collective excitation modes, such as the excitonic or plasmonic state is defined to account for the energy difference between an isolated nanocluster within a large encompassing vacuum region, and the corresponding Bloch state obtained from the propagating LCAO functions.

The binding energy of the collective excitation mode, in this case, denotes the difference between the eigenvalue in each structure relative to the propagator eigenvalue in the reference dielectric system, in which the absorption kick is non-vanishing. We have used Au as the reference system for computing the binding energy of the collective optical excitation mode because only a set of time-dependent polarizations and currents are propagated on near-field scales and the time step used in the simulation is determined by the rate of damping in the material and plasma oscillations and not the speed of light [79].

**Figure 4:**
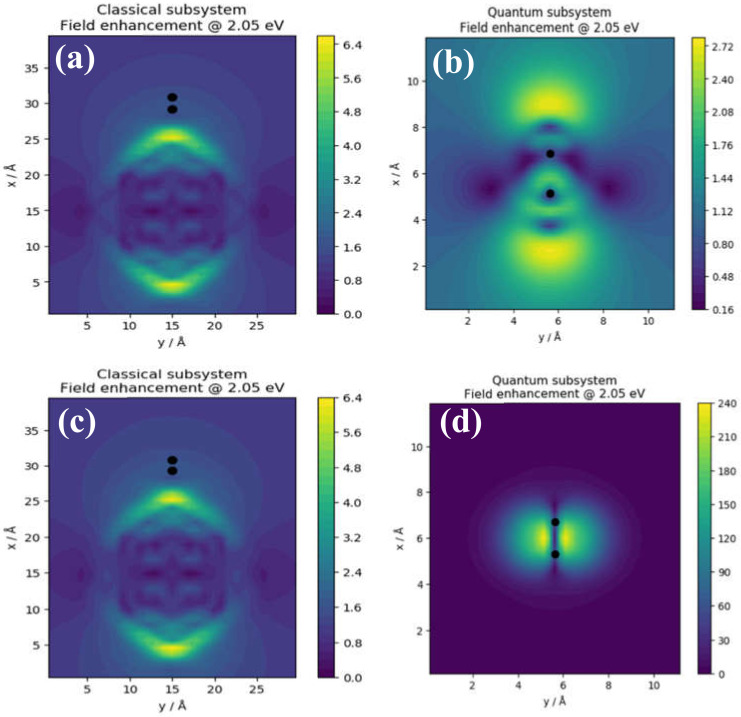
Local distribution of field enhancement in the Classical (**a**,**c**) and Quantum (**b**,**d**) subsystems for TaAs (top panels) and graphene (bottom panels). The color bars denote the field enhancement in Volts/Å.

Figure shows the local distribution of the field enhancement intensity in the classical (Figure 4a,c) and quantum (Figure 4b,d) subsystems in TaAs (top panels) and graphene (bottom panels). These two subsystems represent the nanoparticle and dimer, respectively. It is important to note that the order of magnitude of the field enhancement in the classical subsystem is the same in both TaAs and graphene. This correspondence underscores the equivalence of the renormalized electronic structure as the size of the system is increased. Both the classical and quantum mechanical models reveal fringing field effects although the effect is more noticeable in the region around the dimers. The field intensities are computed at the TQPT point with energy *t* = 2.05 eV. The classical subsystem of the TaAs cluster (Figure 4a) is modeled as a spherical nanoparticle of radius 7.85 Å while the quantum subsystem (Figure 4b) denotes the Ta–As dimer at an interatomic distance of 2.62 Å.

The field enhancements reveal localized regions of high and low intensities around the local geometry—even at a low absorption kick of 10^−5^. Thus, once TQPT has occurred, a change in field intensity does not change the topological order of the electronic phase. Figure 4a,b shows significant enhancements of the field predominantly at the sharp edges (denoted by yellow region) of the nanoparticle geometry. The field lines typically form symmetrical fringes that radiate outwards from the sharp edges in the classical subsystem. Thus, the core of the nanoparticle acts as a sink of the electric field lines. In the quantum subsystem, by contrast, the field enhancement reveals the response of chemical bonds in the Ta–As (Figure 4b) and C–C (Figure 4d) subsystems to the applied external field. Crucially, the field patterns in the two dimers reveal two different responses. We attribute the distorted field profile in (Figure 4b) to the asymmetric charge distribution that exists between Ta and As in the dimer. In Figure 4d, the field profile around the C–C atom is symmetrical. It is thus plausible that this charge symmetry is attributable to the symmetrical response of the local structure around the C–C dimer to the external field. Overall, our analyses reveal that the applied field produces an auxiliary or induced field as a direct response to the changes in the local potential due mostly to the moving carriers. In Section 3.2.2, it is shown that this carrier motion forms a wavelike collective mode akin to charge density waves.

**Figure 5:**
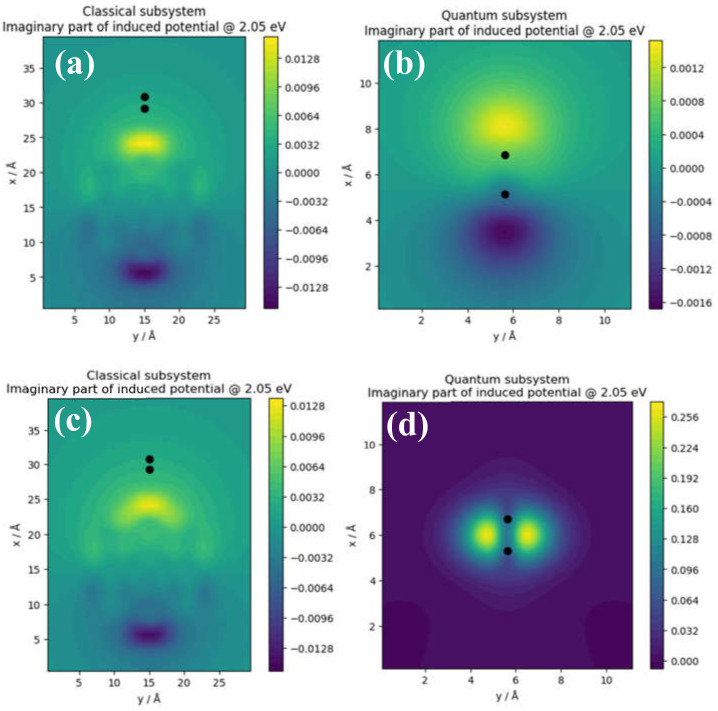
Induced potentials in TaAs (top panels) and graphene (bottom panels) for classical (**a**,**c**) and quantum (**b**,**d**), subsystems. The color bars denote the induced potential in volts.

The electric field is the negative potential gradient that arises because of the potential caused by the induced volume charge density. As such, the local distribution of the induced potential in the topological phase is analyzed in the following paragraphs. Figure 5 shows the induced potential in TaAs (top panels) and graphene (bottom panels), respectively for the classical and quantum subsystems. In the classical subsystems for both TaAs (Figure 5a) and graphene (Figure 5c), the profiles of local potential are similar insofar as that the order of magnitude of the potential and distribution of equipotential surfaces are concerned. However, there are minimal albeit noticeable differences in the distribution of localized regions of high and low potentials. Although the spatial distribution of localized regions of local potential extrema is nearly equivalent in both structures, subtle differences are also observable. This is consistent with the order of magnitude of the field enhancement in the same subsystem.

Figure 5b,d show the imaginary part of the induced potential in the quantum subsystem of the Ta–As and C–C dimers respectively. Firstly, this shows that the induced potential in the Ta–As dimer is three orders of magnitude smaller than the C–C dimer. Secondly, there is a juxtaposition of two adjacent regions of maximum and minimum potential away from the ionic cores of the dimers. Notice that the extremal potentials are shifted along the axis of the Ta–As dimer. The spatial location of the extremal potentials of the C–C dimer is shifted away from the axis of the dimer by 90°. This shift distorts the effective background potential of the dimer. Lastly, since the renormalization of the ground state guarantees equivalence between the two species in models of quantum subsystems (see Figure 5b,d), the origin of the discrepancy is attributable to the induced potential being a short-ranged function of the Ta–As distance in the dimer.

Consider that there is no single near-neighbor distance that characterizes the Ta–As bond length along the dimer axis uniquely. This inherent lack of structural inversion symmetry makes the Ta–As bond length an ill-defined property along the nanoline axis. Thus, the local structure of the TaAs nanoline is challenging to model, unlike the carbon nanoline. Therefore, it is plausible to ascribe the discrepancy observed in the magnitude of the induced potential within the quantum subsystem to the nonexistence of a proper bond length in the Ta–As dimer. These suggest that it is the symmetric distribution of carrier density around ionic cores and the C–C bonds that lead to the substantial increase in the magnitude of the induced volume charge density in graphene. Secondly, although the positions of the local extrema are shifted slightly away from the ion cores, the juxtaposition of two adjacent regions of extremal potentials at alternate lattice sites distorts the effective background potential to modulate carrier transport. In the following subsection, we analyze the volume charge density in the two quantum subsystems to show that despite the discrepancy in the magnitude of the induced potentials in Ta–As and C–C dimers, the order of magnitude of the underlying volume charge density is consistent with the induced potentials in the quantum limit of both materials.

**Figure 6:**
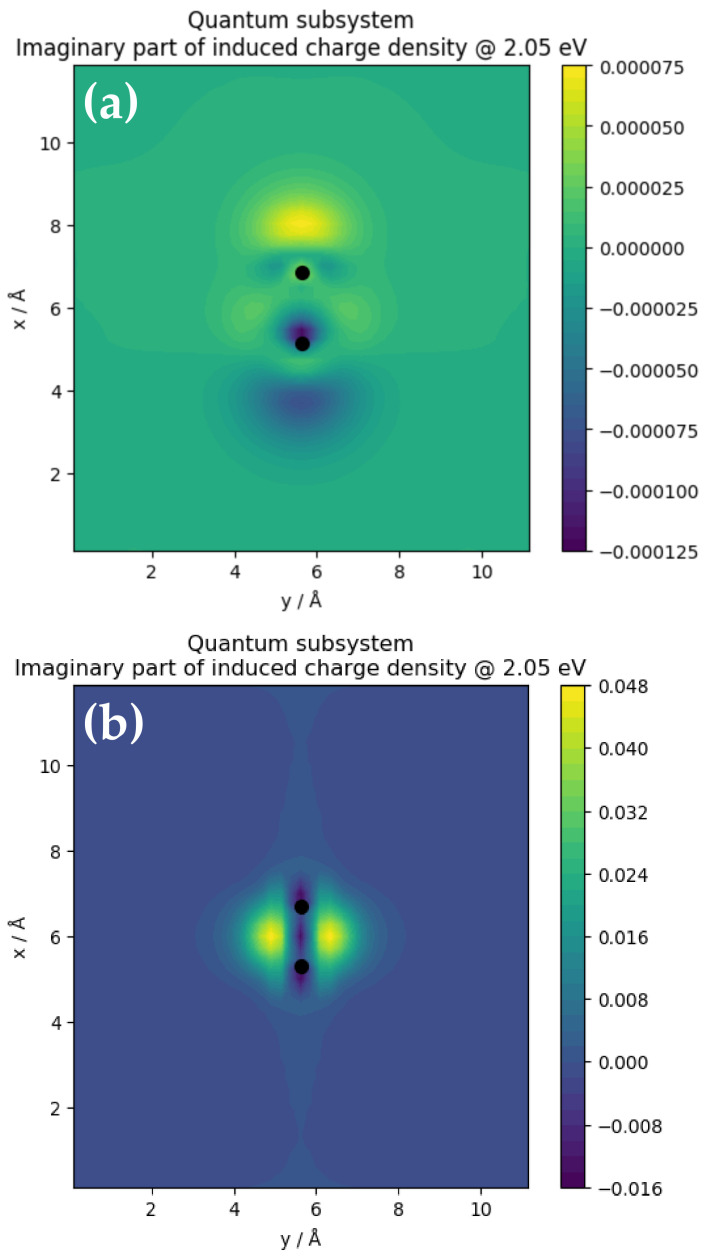
The imaginary part of the induced volume charge density in the quantum subsystems Ta–As (**a**) and C–C (**b**) dimers. The color bars denote the induced volume charge density in Coulombs/Å^3^.

Figure 6 shows the imaginary part of the induced volume charge density in the two quantum subsystems denoted by the Ta–As (see Figure 6a) and C–C (see Figure 6b) dimer. The spatial distributions of charge density reveal pockets of high and low charge density around the ionic sites. For instance, the region between the Ta and As atoms (see Figure 5a) is characterized by a mixture of both high and low charge density whereas in the region between the two C ions of the dimer (see Figure 6a), the low charge density distribution is symmetrical along the axis of the dimer. In both cases, the localized regions of high and low charge densities are embedded in a uniform background of vanishing charge density—especially at large radial distances away from the dimers. Nevertheless, a noticeable oscillation in the charge density distribution is observable around the Ta and As ions of the dimer. By contrast, this is completely absent in the C–C dimer. The magnitude of the charge density localization along the axis of the C–C dimer is low (see Figure 6b). Two localized regions of high charge density are located adjacent to the axis as if their spatial coordinate has been shifted by 90° along with the interatomic distance. Thus, insofar as the regions of charge accretion and depletion are symmetrically distributed around the dimer atoms, a constant induced potential is expected. In both structures, the degree of localization and symmetry of the distribution of spatial regions of the induced charges densities suggest that a finite amount of work must be done on each system to initiate carrier dynamics in the ordered topological electronic phase.

Figure 6 gives an insight into the plasmonic response of the nanoclusters when integrated into a nanojunction. The charge distribution pattern in the classical subsystem is like the quantum subsystem in the sense that the charge density is highest at the vicinity of the ion cores and decreases away from it. These suggest that within the limit in which the length of the Ta–As and C–C dimers are sufficiently large, signatures of the electrodynamic response in both classical and quantum subsystems agree qualitatively. Since the classical and quantum mechanical limits in the above analyses are determined by both atomistic and first principles QSFDTD calculations, the actual electrodynamic phenomenon that culminates in the formation of the induced field is a dynamic process. The distortion in the electrostatic potential background in Dirac materials suggests that the transport of carriers is subjected to the additional potential wells and barriers at the TQPT point. Insights to the resultant time propagation of the carrier density are obtained from the photoabsorption spectra. It is therefore plausible that the external field on the surface of the Dirac material will not necessarily be uniform during the carrier transport at the TQPT point.

Similar studies of the plasmonic response of metallic nanojunctions have suggested that a strong correlation exists between the imaginary part of the induced volume charge density distribution with the excitation frequencies and spectral positions of the dominating resonances of the collective excitation [80]. The plasmonic response of our quantum subsystems agrees with Fukuoka and Tanabe’s attribution of the strong enhancement of the electromagnetic field energy in the region surrounding the nanoclusters to the unique signature of plasmons [81]. Moreover, the established correlation of the plasmonic modes with the quantized carrier transport properties reveals that the mechanism is driven by the lightning-rod effect at the atomic scale [82], which is a signature of the local distribution of field enhancements (Figure 4). Therefore, we expect facile plasmons in multilayer heterostructure systems due to the strong induced potential gradients in the quantum subsystems (Figure 5), which is attributable to the presence of heterobilayer interfaces. We also find in Section 3.2.2 that the collective oscillation of the free electron gas yields a plasmonic response that is akin to the carrier density wave phase.

#### 3.2.2. Optical Photoabsorption

**Figure 7:**
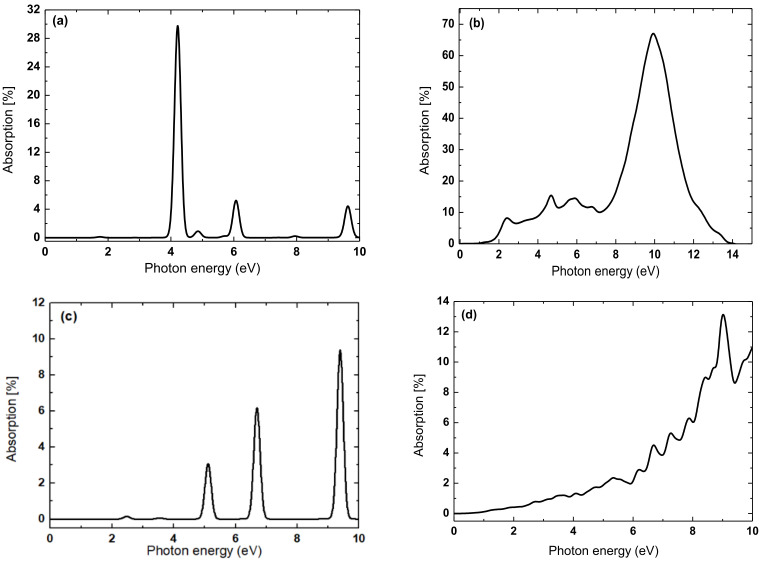
Structure dependence of optical photoabsorption spectra. Top panels show the spectra of the sp^3^-hybridized C–C dimer (**a**) and the carbon nanoline (**b**). Bottom panels show the absorption spectrum for the Ta-As dimer (**c**) and the TaAs nanocluster (**d**).

One of the established ways for linking the results of computational studies of field-theoretical predictions with experimental measurements is to study the optical spectra [83]. Figure 7 shows the structural dependence of the optical photoabsorption spectra after Gaussian folding at a width of 0.1 eV. The top panels (Figure 7a,b) denote the photoabsorption spectrum in the C-C dimer and bulk structures of graphene while the equivalent spectra for TaAs are shown in the bottom panels (Figure 7c,d). The photon absorption gives the fraction of the incident radiation that is absorbed by the material over a range of photon energies. The discrete peaks in (Figure 7a,c) show that optically induced interband transitions can also occur between valence and conduction bands states in a manner akin to the quantum dots. In (Figure 7b,d) the absorption spectra of the two bulk structures are broad with a dominant peak at 9.96 eV (TaAs) and 8.9 eV (graphene), respectively.

The dimers are characterized by well-resolved peaks with principal absorption at energies 4.2 eV (graphene) and 9.4 eV (TaAs). These suggest that the C–C dimer absorbs radiation strongly at low energy of 4.2 eV compared to the relatively low absorption of graphene carbon dimers at 9.4 eV. The poor resolution of the dominant cluster peaks in the bulk photoabsorption spectra is an intriguing non-linear optical effect. Moreover, the position of the dominant peak in the spectra of both bulk structures is well-resolved in the cluster spectra. However, they both appear as low-intensity peaks but are more diminished in TaAs than graphene. We attribute the poor resolution of optical absorption peaks in the bulk to the quantum confinement-induced broadening of the degree of freedom of electronic state space of the bulk structure. It is plausible that the reverse effect is responsible for the sensitivity of the size of the bandgap to the nanoparticle size.

Despite the underlying chemical differences between the graphene and TaAs, it is important to recall that their zero-field ground state has a gapless electronic structure. However, the semiconducting phase emerges from the suitable combination of perturbation fields (see Section 3.1). With a finite gap between their valence and conduction bands, transport in the semiconducting phase supports charge carrier transitions between the electron (*e*) and hole (*h*) states. The first absorption maxima in (Figure 7a–d) represent the transition of electrons from the valence band to the conduction band leaving behind a hole, which can combine with an electron to produce an exciton. The photon energy of the first absorption maximum approximately yields the size of the bandgap. Where the material hosts bound *e-h* pairs (i.e., excitons), the discrete peaks in (Figure 7a,c) suggest that additional transitions from the bound electron-hole pairs must occur. This assertion is also valid because the C-C dimers show small absorption peaks at 1.74 and 7.96 eV in (Figure 7a), and at 2.44 and 3.68 eV in (Figure 7c).

**Figure 8:**
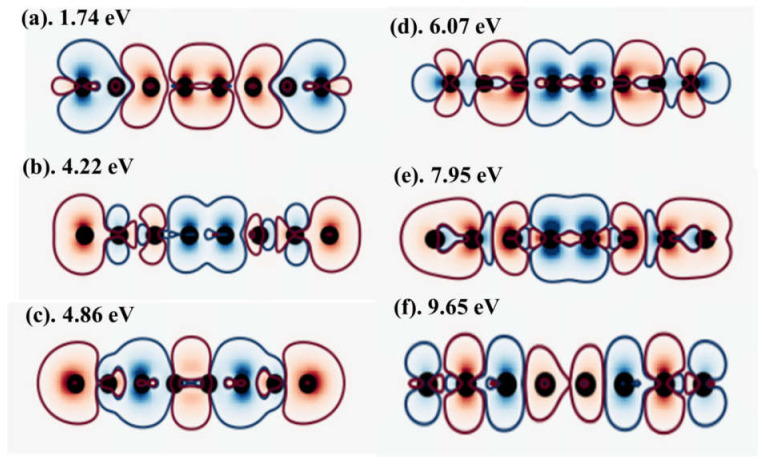
Field-induced carrier density wave along the carbon nanoline at a photon energy of 1.74 (**a**). 4.22 (**b**). 4.86 (**c**). 6.07 (**d**). 7.95 (**e**). 9.65 eV (**f**). Black balls denote atomic cores, while blue and red colors indicate minimal and maximal carrier density (in Coulombs/Å3), respectively.

Figure 8 shows the field-induced carrier density wave along the carbon nanoline at the photon energies corresponding to distinct absorption peaks in the spectra. This shows that the induced field is distributed symmetrically around atoms of the nanoline, with alternating domains of high and low intensities. We note that the field intensity is distributed in a characteristic manner wherein the alternating regions of low (red) and high (blue) intensities alternate. This indicates the formation of a unique non-linear optical signature at the TQPT point akin to a quantum fluid-like phase. The induced electric field sets up as a standing wave like normal modes of vibration. Their localization intensities change periodically as the energy of the absorbed photon increases. At increasing photon energy of 1.74 to 7.95 eV (see Figure 8a–e), the halos of the low field are localized around the two carbon atoms cores located at the center of the nanoline.

A somewhat similar trend is observed at 9.65 eV (see Figure 8f), except that the high field halos are independently localized on the two C atoms at the center of the nanoline. We ascribe the wave-like induced charge density in the bond region and around near-neighbor sites to the formation of static carrier density waves. This offers potential grounds for rich physics and emergent applications in photonics, optoelectronics, and quantum computing. The static induced charge density denotes the charge density wave (CDW) phase. In nanoline systems in which time-reversal symmetry is broken, it corresponds to the spin density wave (SDW) phase. The same trend is found in the Ta–As dimer because the induced charge density is localized around constituent atoms despite its isolated nature. Carriers transported via the CDW phase form a standing wave pattern and can carry electric current collectively. Thus, dissipation-free transport is achievable when carrier current flows through this topologically ordered quantum fluid phase.

**Figure 9:**
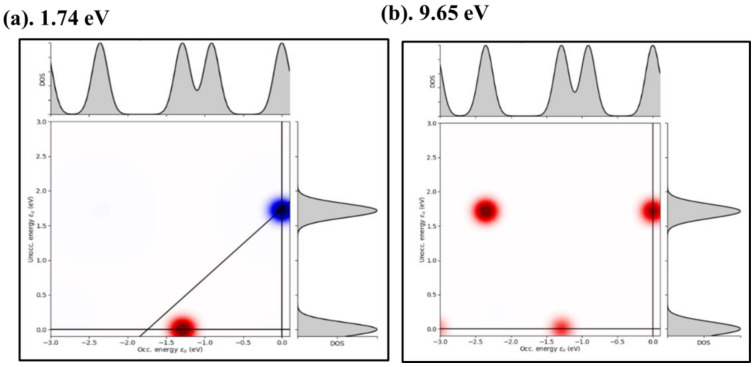
Combined electronic density of states (in states/eV.cell) and the optical transition contribution map at extremal energies 1.74 eV (**a**). 9.65 eV (**b**). In the photon absorption spectrum of the graphene system. Blue and red colors indicate net deficit and surplus of electron density, respectively.

Figure 9 shows the combined electronic DOS and the optical transition contribution map (TCM) at extremal energies in the photon absorption spectrum of the graphene system. In addition to the TCM, (Figure 9) shows two independent DOS—one each for total electronic states (top spectra) and the unoccupied (i.e., hole) states (right spectra) for photon absorption peaks the 1.74 eV (Figure 9a) and 9.65 eV (Figure 9b). Firstly, the spectra of the total electronic DOS and the *h* states are invariant under changes in photon absorption energy showing that the density of states of the carrier species does not depend on the photon absorption. Secondly, analysis of the TCM reveals that each peak in the absorption spectra consists of many single-particle *e-h* transitions. Holes are created from the Fermi level (*i.e*., 0.0 eV) down to the top of the occupied valence band states about −3.0 eV. In addition, carriers are created from states in the lowest unoccupied conduction band up to about 1.75 eV. In this broad energy range, only carbon *p*- states are active and the total transition shows no mixing with contributions from the carbon *2s*-states. Lastly, the number of *h* states is higher at 9.65 eV compared to 1.74 eV. For the low-energy absorption, there is a net deficit of electron density at the Fermi level while there is a surplus at 1.75 eV. However, the TCMs in (Figure 9) do not show any collective dipolar oscillations since the surplus density disappears at 9.65 eV.

The validity of the above analyses stems from our computation of the discrete Fourier transform of the reduced density matrix in the frequency domain. This is crucial because the frequency domain analysis and their Fourier transforms underpin the engineering of electrical transport signals and systems. For instance, a real-space discrete Fourier transform of a carrier transport signal at fixed bias constitutes an input that has no variation and, therefore, has the only slowest, constant Fourier component, which corresponds to the direct current, component. This is equivalent to the mean of the carrier transport signal. In addition, the time propagation scheme for the perturbed ground state electronic density yields additional insights on the local structure, as well as the ability to distinguish between peaks in the absorption spectrum that correspond to specific principal directions in a lattice. Since the dipole moment is generated from displacements in the charge density, the strong peaks in the absorption spectrum signify nearly harmonic oscillations in the charge density, and understanding these dynamic phenomena requires sensitive probes that respond to spin, charge, and orbital degrees of freedom of carriers which encompasses the chiral edge states under excitation from the dipole field.

## 4. Conclusions

In summary, we have performed field-theoretical computations based on the combination of first-principles calculations with time-dependent density functional theory to study the carrier transport phases that emerge from the optical excitation of the electronic structure in TaAs and graphene as prototypical models of Dirac materials. The dynamic properties of the topologically ordered carrier transport phases are unraveled in terms of the field-induced modifications of the electronic structure under the renormalization constraint from the drive field. We find distinctive features of plasmonic modes as an emergent response of the topological quantum phases. Our results reveal unique critical points in the topological phase diagram as a function of the external drive fields. We find that mixing of two or more internal and external fields in the tight-binding model of the tuned graphene model generates a rich spectrum of non-trivial phases, and this provides the theoretical basis for experimental platforms to tune the carrier transport states of Dirac materials using internal and external fields. This study offers insights into the near field regime of quantum electrodynamics for carriers in topological quantum materials, and on how their quantum interferences are tractable in the dynamics of free carriers. Our results reveal strategies for obtaining topological band structures from the engineering of trivial equilibrium bands using electromagnetic fields. The insights gained herein underpin the physical bases for strategies to develop adaptive material platforms that are suitable for emergent applications in plasmonics, optoelectronics and photonics.

## Data Availability

The data presented in this study are available in the article.
